# Effects of Hypocretin/Orexin Cell Transplantation on Narcoleptic-Like Sleep Behavior in Rats

**DOI:** 10.1371/journal.pone.0095342

**Published:** 2014-04-15

**Authors:** Oscar Arias-Carrión, Eric Murillo-Rodríguez

**Affiliations:** 1 Unidad de Trastornos del Movimiento y Sueño (TMS), Hospital General Dr. Manuel Gea González, Mexico City, Mexico; 2 Unidad de Trastornos del Movimiento y Sueño (TMS), Hospital General Ajusco Medio, Mexico City, Mexico; 3 Laboratorio de Neurociencias Moleculares e Integrativas, Escuela de Medicina, División Ciencias de la Salud, Universidad Anáhuac Mayab, Mérida, Yucatán, Mexico; Universitätsklinikum Carl Gustav Carus an der Technischen Universität Dresden, Germany

## Abstract

The sleep disorder narcolepsy is now considered a neurodegenerative disease because there is a massive loss of neurons containing the neuropeptide hypocretin/orexin (HCRT). In consequence, narcoleptic patients have very low cerebrospinal fluid (CSF) levels of HCRT. Studies in animal models of narcolepsy have shown the neurophysiological role of the HCRT system in the development of this disease. For example, the injection of the neurotoxin named hypocretin-2-saporin (HCRT2/SAP) into the lateral hypothalamus (LH) destroys the HCRT neurons, therefore diminishes the contents of HCRT in the CSF and induces narcoleptic-like behavior in rats. Transplants of various cell types have been used to induce recovery in a variety of neurodegenerative animal models. In models such as Parkinson's disease, cell survival has been shown to be small but satisfactory. Similarly, cell transplantation could be employed to implant grafts of HCRT cells into the LH or even other brain regions to treat narcolepsy. Here, we report for the first time that transplantation of HCRT neurons into the LH of HCRT2/SAP-lesioned rats diminishes narcoleptic-like sleep behavior. Therefore, cell transplantation may provide an effective method to treat narcolepsy.

## Introduction

Narcolepsy is a debilitating neurological disease characterized by overwhelming daytime sleepiness, loss of muscle tone without impairment of conscience (named cataplexy), and premature transitions from waking to rapid eye movement sleep (REMS) [1]–[4]. The assumption that narcolepsy is related to the hypocretin system function (HCRT-1 and -2, also known as orexin-A and -B, respectively) is supported by several lines of evidence. First, these neuropeptides are synthesized in the hypothalamus [4]–[6] and project to major arousal centres; second, the HCRT system disruption induces behavioral narcoleptic symptoms observed in experimental models [7]–[10]; third, clinical studies in narcoleptic patients show undetectable levels of HCRT in the CSF [11]; and finally, *post-mortem* studies reveal a loss of HCRT-immunoreactivity as well as mRNA [12], [13].

Experiments on the HCRT/ataxin-3 narcolepsy mouse model demonstrated that intracerebroventricular (ICV) injections of HCRT-1 can compensate for HCRT cell loss and reverse most of the narcolepsy symptoms including cataplexy [14]. However, ICV injections in humans are not feasible and thus John and colleagues [15] investigated intravenous (IV) application methods. On the other hand, the size of both HCRT peptides suggests that they would be unlikely to penetrate the blood brain barrier (BBB) [16]. As predicted, even high HCRT-1 concentrations in the blood stream did little to raise HCRT levels in the brain. This problem was overcome by Mieda and co-workers in a mouse model designed to drive non-regulated over-expression of the HCRT gene in mice lacking HCRT neurons, resulting in a rescue from narcolepsy symptoms [14]. Instead, it seems that bypassing the BBB might provide an easier and less invasive way to increase HCRT signalling in the brain. This could be achieved by engineering HCRT analogues or HCRT receptor agonists that are small enough to pass through the pores of the BBB. However, thus far efforts to design either a HCRT-1 analogue or a receptor agonist of the appropriate size have failed (see review [17]). A simple and elegant strategy to bypass the BBB may be to increase HCRT signalling by intranasal delivery of HCRT-1 [18]. Recent studies have shown that this route of application does not rely on blood transport and bypasses the BBB and delivers the HCRT peptide directly into the brain [19]. Alternative treatment strategies for narcolepsy may include viral delivery of a transgene to drive HCRT expression in non-HCRT cells [20]. So far this approach has shown promising results in mice, as Liu and colleagues demonstrated a rescue from narcolepsy symptoms through non-specific overexpression of HCRT by non-HCRT cells in the hypothalamus in two different animal models of narcolepsy [21], [22].

Thus, the findings that HCRT neurons are lost in narcolepsy lead to the hypothesis that these neurons might be replaced [3], [4], [10], [23]–[25] and raise the question whether the transplantation of HCRT neurons into LH of lesioned rats would improve the sleep disturbances in a narcoleptic experimental model. Therefore, our purpose was to test the hypothesis that HCRT-cell transplantation might decrease the sleepiness in the narcoleptic-like model.

## Materials and Methods

Male Wistar rats (250–300 g) were bred at the facilities of our Institute and efforts were made to minimize animal suffering, and to reduce the number of animals used. All procedures were conducted in accordance with the guidelines of the Mexican Institutes of Health Research (DOF. NOM-062-Z00-1999) as well as the National Institutes of Health Guide for the Care and Use of Laboratory Animals (NIH publication No. 80–23, revised 1996) and the experimental protocol was approved by the Institutional Animal Care and Use Committee (IACUC), Universidad Anáhuac Mayab (FM/2010-2A).

All procedures that involved discomfort, distress, pain as well as injury were minimized through the use of anaesthesia as well as analgesic drugs. Use of analgesic, anaesthetic, and tranquilizing drugs and/or comfortable restraining devices, were included to minimize discomfort, distress, pain, and injury such as acepromazine, xylazine, and ketamine (cocktail of acepromazine (0.75 mg/kg), xylazine (2.5 mg/kg), and ketamine (22 mg/kg), im (0.05 ml), cefazolin (20 mg/kg, im). Adittionally, a post-operative pain reliever bupernorphine (0.1 mg/kg, im, as needed) was also employed. Heating blankets were used for thermal comfort for the rats during surgery. To ensure the general well-being of the animals, rectal temperature was also monitored. As a post-operative support, animals were placed in heating pads and blankets. The criteria by which a decision to euthanize a rat post-operatively was based on a remarkable decrease in weight, hypophagia and water intake. The suggestions from the veterinary staff who monitored the conditions of the rats during the entire project were considered for the decision. On the other hand, animals that showed signs of severe suffering, pain or disease were sacrificed with a lethal dose of pentobarbital (IM). All bodies were discarded according to the Mexican guideline (NOM/GCUAL) as well as the National Institutes of Health Guide for the Care and Use of Laboratory Animals (NIH publication No. 80–23, revised 1996) standards. To prevent disease, necessary arrangements for the diagnosis and treatment of any medical abnormality were considered.

### Experiment 1. Experimental model of narcolepsy using HCRT2/SAP

Under aseptic conditions and deep anaesthesia (acepromazine [0.75 mg/kg], xylazine [2.5 mg/kg], and ketamine [22 mg/kg, i.p]) animals were placed into the stereotaxic frame instrument (Kopft Instruments) and a cannulae-injector was aimed to the LH (Bregma: A = −3.3; L = ±1.6; H = −8.2 mm). Rats received bilaterally an injection of either saline (n = 5) or HCRT2/SAP (n = 5; 490 ng/0.5 µL. Advanced Target Systems. CA, USA). This procedure was conducted as described previously [26]–[28]. The microinjections were delivered manually using a 5 µL Hamilton microsyringe (Hamilton Co. Reno, NV, USA) attached to an injector *via* tubing (FEP Teflon tubing: 0.65 mm outside diameter ×0.12 mm internal diameter. BAS, West Lafayette, IN. USA). The administrations were carried out manually (flow rate: 0.5 µL/min) with the injector left in the target for an additional 10 s to ensure extrusion from the tip, and to minimize distribution of treatments upwards on the cannulae. After surgery, rats were housed singly in Plexiglass cages under constant temperature (21±1°C) and a controlled light-dark cycle (lights on: 07:00–19:00 h; 150 lux during the lights-on period). Food and water were provided *ad libitum*. Twenty-one days after the surgery, control and lesioned rats were deeply anesthetized with pentobarbital (150 mg/kg i.p.) and perfused transcardially with 0.9% saline (50 mL) followed by 500 mL of phosphate-buffered 4% paraformaldehyde (pH 7.0). The brains were removed, post-fixed overnight in formalin, and then equilibrated in 30% sucrose (0.1 M PBS), and stored at 4°C. Brains were dissected (1∶5 series, 30 µm) on a freezing microtome and stored in PBS-azide (4°C). One series from each brain of coronal brain sections was stained for HCRT as previously described [23], [24]. One person blind to the experiment counted all of the immunoreactive somata on both hemispheres (control = 5; lesion = 5) using a 10 square grid (50 µm/square) centered on the fornix. At least 12 sections (1∶5 series) that encompassed the rostral pole of the LH to the caudal pole of the tuberomammillary nucleus (TMN) were examined (sections between −1.3 and −1.4 mm referred to Bregma).

### Experiment 2. Sleep profile in HCRT2/SAP lesioned rats

Under aseptic conditions and deep anaesthesia as described in Experiment 1, a different group of animals received a bilateral injection into LH (Bregma: A = −3.3; L = 1.6; H = −8.2) of either saline (control, n = 3) or HCRT2/SAP (n = 4; 490 ng/0.5 µL; Advanced Target Systems. San Diego, CA. USA). Additionally, animals were implanted with electrodes to record the electroencephalogram (EEG) and electromyogram (EMG). Briefly, the EEG electrodes (Plastics One. Roanoke, VA. USA) consisted of four miniature screws, two of which were placed on either side of the sagittal sinus but over the occipital cortex, and the other two EEG electrodes were placed on either side of the sagittal sinus over the frontal cortex. For the EMG signal, two flexible wire electrodes were inserted bilaterally into the nuchal muscles of the rats. The electrodes were placed and secured onto the skull using dental cement. Right after the surgeries, the animals were attached to the sleep recording cable and placed singly into the sleep-recording chambers. Throughout the experiment the rats had food and water available *ad libitum* and had a controlled environment under a constant temperature (21±1°C) and a controlled light-dark cycle (lights on: 07:00–19:00 h; 150 lux during the lights-on period) for 21 days.

### Experiment 3. Sleep profile in HCRT2/SAP lesioned rats that received HCRT neurons grafting

In this experiment, a new group of rats received a bilateral injection into LH as described in Experiment 1. Electrodes to record the EEG and EMG were used as described in Experiment 2. Right after surgery, the rats were attached to the sleep recording cable and placed singly into the sleep-recording chambers with food and water available *ad libitum* and controlled environment under a constant temperature (21±1°C) and a controlled light-dark cycle (lights on: 07:00–19:00 h; 150 lux during the lights-on period) for 21 days. On day 22^nd^ post-lesion, the HCRT neurons transplants were performed into both, the control and lesioned groups. Cell preparation as well as transplantation technique were carried out as previously published [23], [24]. Briefly, Wistar pups (8–10 days old) were decapitated and the brain removed rapidly and placed into a plastic matrix for pups brains that facilitated dissection. The matrix was immersed in ice-cold artificial-CSF with O_2_ flux. Then, a slice of the brain was obtained and with the aid of a dissecting microscope the LH was dissected. Bilateral samples obtained from 3 pups in the culture medium (Dulbecco's Modified Eagle Medium: DMEM; GIBCO. Life Technologies. Grand Island, NY. USA) were pooled and, this samples were considered as final tissue samples. The fragments were dissociated enzymatically in Spinner's saline solution with 2 mg/mL of collagenase (Worthington, type I. Sigma-Adrich Co. St Louis, MO. USA) and 15 mg/mL of deoxyribonuclease (Type II. Sigma-Adrich Co. St Louis, MO. USA) for 45 min at 37°C. The dissociated cells were kept in culture medium and supplemented with 4.5 µg insulin (Sigma-Adrich Co. St Louis, MO. USA), 100 U/mL penicillin (Sigma-Adrich Co. St Louis, MO. USA), 100 mg/mL streptomycin and 2.5 mg/mL fungizone [GIBCO. Life Technologies. Grand Island, NY. USA]). The tissue was centrifuged (600 rpm for <2 min) and the cell preparation for transplants was carried out as previously reported [23], [24]. One pooled sample was grafted to one host. Twenty-two days after the lesion, control and lesioned animals with the EEG/EMG electrodes were anesthetized and placed back into the stereotaxic frame. Both groups of animals (control and lesioned) received bilaterally a suspension of cells injection into the LH (4 µL; flow rate of 0.5 µL/min) containing HCRT neurons which were obtained previously from the LH of the rat pups as described in Experiment 3. Right after the grafting surgery, control and lesioned groups were placed again into the sleep chambers and attached to the sleep recording system during 21 days. The sleep data consisted in the analysis of the EEG/EMG signals in the whole study. The EEG signal was obtained from two contralateral electrodes (frontal–occipital) and the EMG signal from the nuchal muscles. The EEG/EMG signals were recorded using a Grass Model 12 polygraph, filtered (EEG = 0.3–30.0 Hz, EMG = 0.3–1.0 kHz), digitized (sampling frequency = 128 Hz; National Instruments) and stored on a computer with the aid of a data acquisition software (ICELUS). The EEG and EMG data was scored in 12 s intervals for wakefulness (W), slow wave sleep (SWS) and REMS with the aid of a sleep-scoring program (ICELUS). Sleep stages were classified visually as follows: The presence of low amplitude-high frequency in the EEG/EMG activity displaying high values, and alpha power spectra showing higher values was the criteria to identify W. Next, SWS was characterized by high amplitude-slow frequency in the EEG activity which was coupled to low EMG activity and high delta power compared to W, and REMS were identified by the presence of regular theta activity in the EEG signal, and a low to absent EMG signal relative to SWS and W. This criterion was previously reported by our group [29], [30]. Sleep scoring was carried out by a single examiner blinded to the treatment of the animals. Finally, in order to avoid experimental bias, at the end of the immunohistochemical and behavioral studies the code was broken to reveal the treatments of each rat. The statistical analyses of the data were performed using the StatView program (version 5.0.0, SAS Institute. U.S.A.). Data was represented as mean ± standard error of the mean and statistical differences were determined by a Student's *t*-test. Values of *P*<0.05 were considered as statistically significant.

## Results

**Figure 1:**
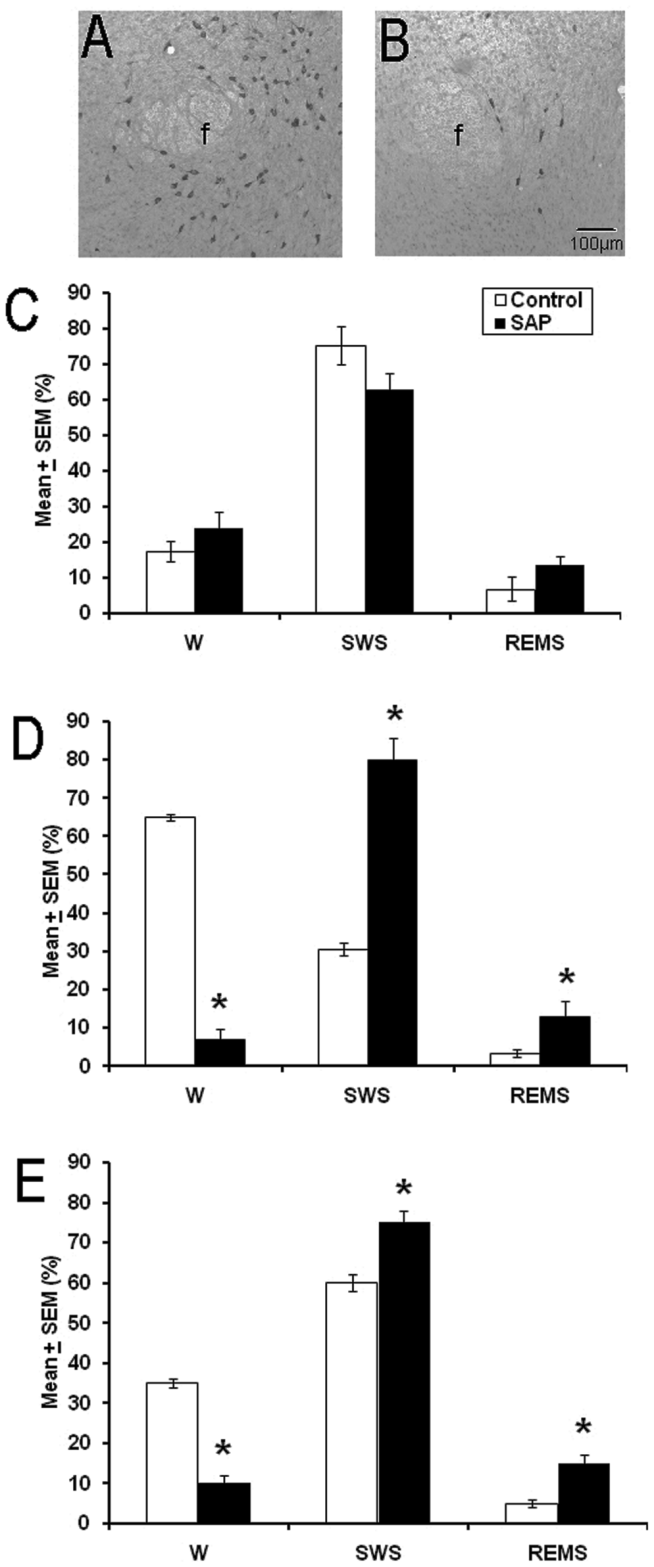
HCRT immunohistochemistry at 21 days post-intrahypothalamic injection of either saline solution (Panel A) or HCRT2/SAP (Panel B). Effects on wakefulness (W), slow wave sleep (SWS) and rapid eye movement sleep (REMS) at 21 days post-intrahypothalamic administrations of either vehicle (control, white bars) or HCRT2/SAP (black bars) during the lights-on (Panel C), lights-off period (Panel D) or the 24 h total time (Panel E). Note that HCRT loss induces a decrease in W and enhances SWS and REMS during the lights-on period as well as in the total sleep time (mean ± SEM of total time of recording [%]; * *vs* control, p<0.05). Abbreviations: f: fornix. Scale bar, 100 µm.

As shown in **Fig. 1**, the immunohistochemical study revealed the presence of HCRT neurons in the LH in a control animal (Panel A); however, the injection of HCRT2/SAP decreased the number of HCRT neurons (Panel B; Control: 1369.1±118.9 vs HCRT2/SAP: 119.43±12.6; df = 8, *t* = 3.427, *p*<0.005). Importantly, degenerative morphological structures were observed in the HCRT2/SAP-treated rats. These findings are in accordance with our previous studies [23], [24], [26]–[28].

As can be seen in **Fig. 1C**, at 21 days post-lesion, the HCRT2/SAP toxin did not induce statistical differences during the lights-on period on W compared to the control group (df = 5; *t* = −0.961; *p* = 0.3), SWS (df = 5; *t* = 1.709; *p* = 0.14) or REMS (df = 5; *t* = −1.470; *p* = 0.20). However, during the lights-off period we observed that the administration of the HCRT2/SAP decreased W (df = 5; *t* = 15.583; *p*<0.0001) and increased SWS (df = 5; *t* = −5.741; *p*<0.002) as well as REMS (df = 5; *t* = −2.437; *p*<0.05; Figure 1D) compared to the control group. Total sleep time (24 h) analysis showed that the HCRT2/SAP lesioned rats displayed a significant decrease in W (df = 5; *t* = 6.569; *p*<0.002) and an increase in SWS (df = 5; *t* = −3.188; *p*<0.02) and REMS (df = 5; *t* = −2.843; *p*<0.03; **Fig. 1E**) compared to the control group. The effects on sleep after the injection of HCRT2/SAP observed in the current study are consistent with previous reports [26], [27].

**Figure 2:**
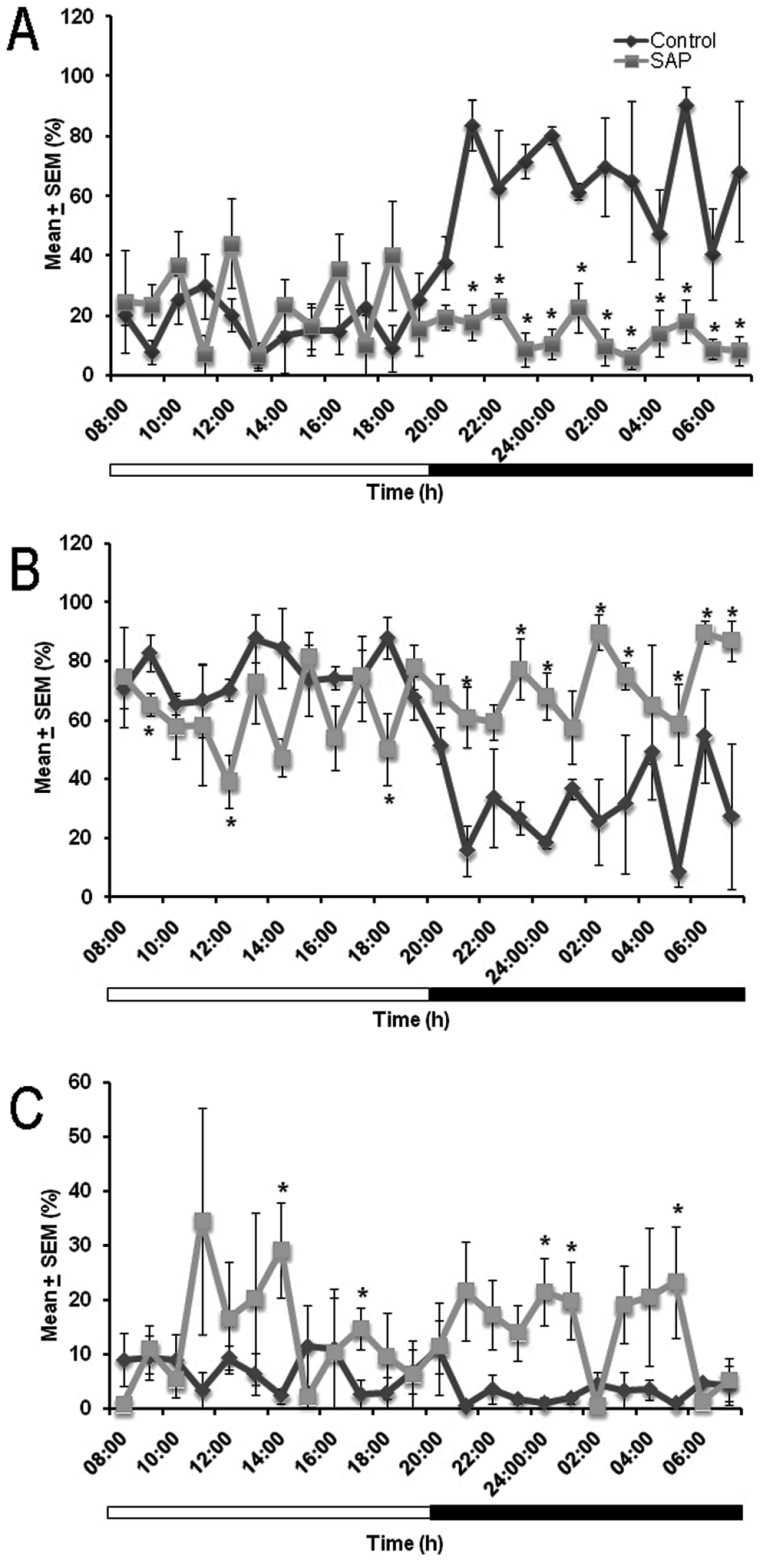
The hourly data at 21 days post-lesion for waking (W) (Panel A), slow wave sleep (SWS; Panel B) and rapid eye movement sleep (REMS; Panel C) after the intrahypothalamic injection of either saline solution (control) or HCRT2/SAP show that the loss of HCRT neurons diminishes W during the lights-on period but increases SWS during the lights-off period. The white bars represent the lights-on period and the black bars indicate the lights-off period (mean ± SEM of total time of recording [%]; * *vs* control, p<0.05).

The hourly analysis of sleep stages across 24 h at 21 days post-lesion showed that the bilateral injection of HCRT2/SAP into the LH decreased W (**Fig. 2A**) during the dark phase compared to the control group. For instance, the effects were observed at the following hours: 14^th^ (df = 5; *t* = 6.608; *p*<0.001), 15^th^ (df = 5; *t* = 2.321; *p*<0.05), 16^th^ (df = 5; *t* = 7.530; *p*<0.0007), 17^th^ (df = 5; *t* = 10.620; *p*<0.0001), 18^th^ (df = 5; *t* = 3.861; *p*<0.01), 19^th^ (df = 5; *t* = 3.864; *p*<0.01), 20^th^ (df = 5; *t* = 2.566; *p*<0.05), 21^st^ (df = 5; *t* = 2.123; *p*<0.05), 22^nd^ (df = 5; *t* = 7.437; *p*<0.0007), 23^rd^ (df = 5; *t* = 2.345; *p*<0.05), and the 24^th^ (df = 5; *t* = 2.913; *p*<0.03). On the other hand, the hour by hour analysis of SWS (**Fig. 2B**) showed changes among control and lesioned group at the 2^nd^ (df = 5; *t* = 2.532; *p*<0.05), 5^th^ (df = 5; *t* = 2.763; *p*<0.03), 11^th^ (df = 5; *t* = 2.389; *p*<0.05), 14^th^ (df = 5; *t* = −3.219; *p*<0.02), 16^th^ (df = 5; *t* = −3.824; *p*<0.01), 17^th^ (df = 5; *t* = −5.185; *p*<0.003), 19^th^ (df = 5; *t* = −4.499; *p*<0.006), 20^th^ (df = 5; *t* = −2.113; *p*<0.05), 22^nd^ (df = 5; *t* = −3.011; *p*<0.02), 23^rd^ (df = 5; *t* = −2.504; *p*<0.05), and 24^th^ (df = 5; *t* = −2.677; *p*<0.04) hours. Finally, the hourly analysis of REMS (**Fig. 1C**) showed that compared to the control group, lesioned animals displayed effects at the following hours: 7^th^ (df = 5; *t* = −2.596; *p*<0.04), 10^th^ (df = 5; *t* = −2.352; *p*<0.05), 17^th^ (df = 5; *t* = −2.783; *p*<0.03), 18^th^ (df = 5; *t* = −2.105; *p*<0.05), and 22^nd^ (df = 5; *t* = −1.842; *p*<0.05). The somnolence-like behavior induced by the HCRT2/SAP observed in the present study is consistent with previous reports [9], [26]–[28].

**Figure 3:**
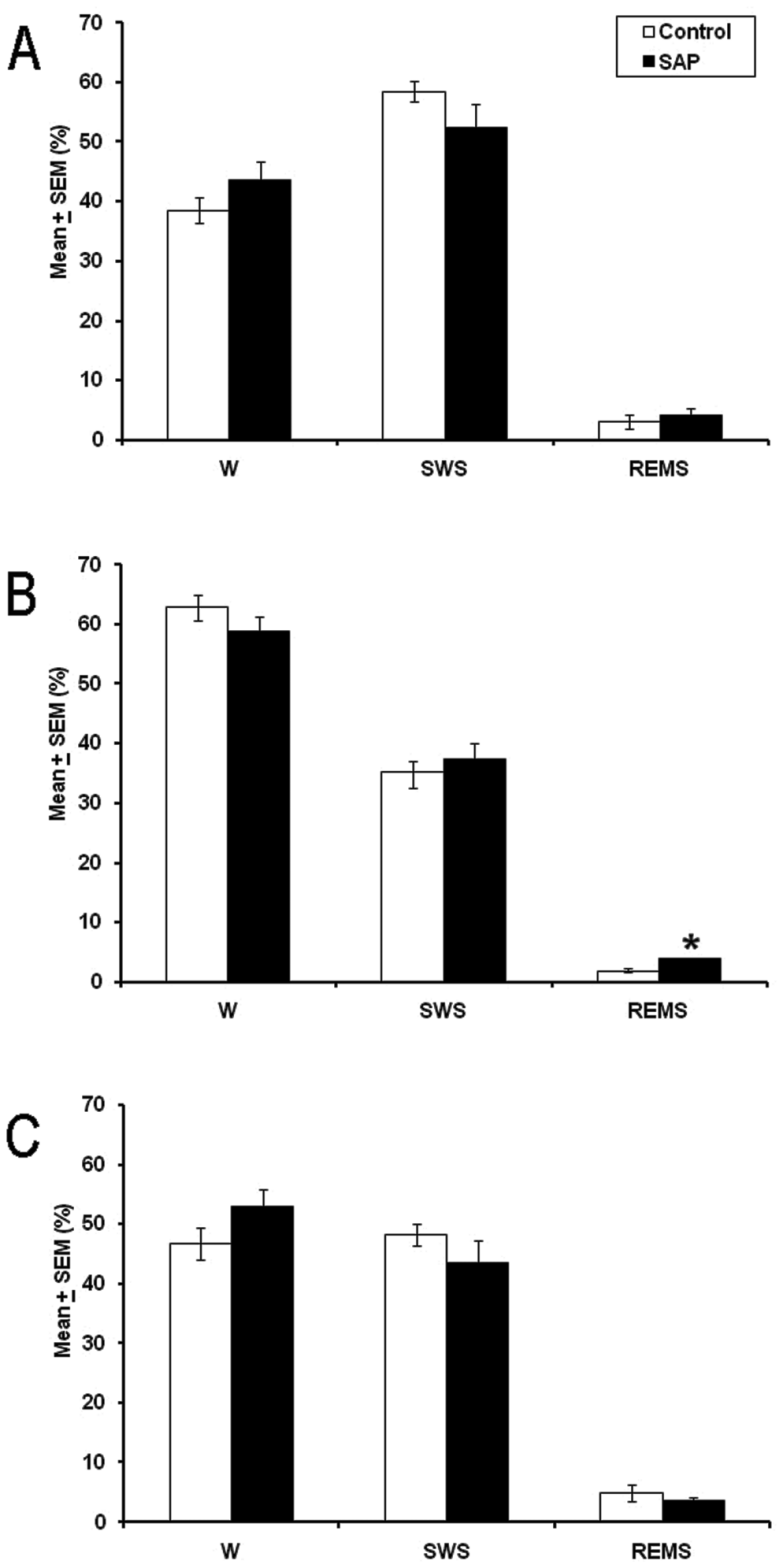
Effects on wakefulness (W), slow wave sleep (SWS) and rapid eye movement sleep (REMS) after 21 days post-transplant of HCRT neurons into LH of saline (white bars) or HCRT2/SAP lesioned rats (black bars) during the lights-on period (Panel A), lights-off period (Panel B) or the 24 h total time. Note that the HCRT grafting improved the sleep-wake cycle in lesioned rats (Panel C; mean ± SEM of total time of recording [%]; * *vs* control, p<0.05).

In the final experiment, we examined whether the sleep disturbances in the HCRT2/SAP lesioned rats were improved through a transplant. Twenty-one days post-lesion, control and lesioned animals received HCRT grafts into the LH and sleep recordings were obtained during the following 21 days. **Fig. 3A** shows that at the 21^st^ day post-graft, the sleep parameters during the lights-on period had no statistical differences among both groups: W (df = 14 *t* = −1.386; *p* = 0.19), SWS (df = 14 *t* = 1.582; *p*< = 0.13) and REMS (df = 14; *t* = −0.574; *p* = 0.5). In addition, the analysis of the sleep stages during the lights-off period showed no significant differences on W (df = 14 *t* = 1.266; *p* = 0.2) and SWS (df = 14 *t* = −0.632; *p* = 0.5). However, HCRT2/SAP lesioned rats that received HCRT transplant showed an enhancement in REMS compared to control group (df = 14 *t* = −3.418; *p*<0.004; **Fig. 3B**). Although that lesioned animals received HCRT graft, total sleep time (24 hr) analysis (**Fig. 3C**) exhibited no statistical differences compared to control group which also received HCRT transplant: W (df = 14; *t* = −1.501; *p* = 0.1), SWS (df = 14; *t* = 1.235; *p* = 0.2) and REMS (df = 14; *t* = 0.826; *p* = 0.4). The results suggest that the transplant of HCRT cells indeed improved the sleep disturbances caused by the lesion of HCRT neurons using HCRT2/SAP injected bilaterally into the LH.

**Figure 4:**
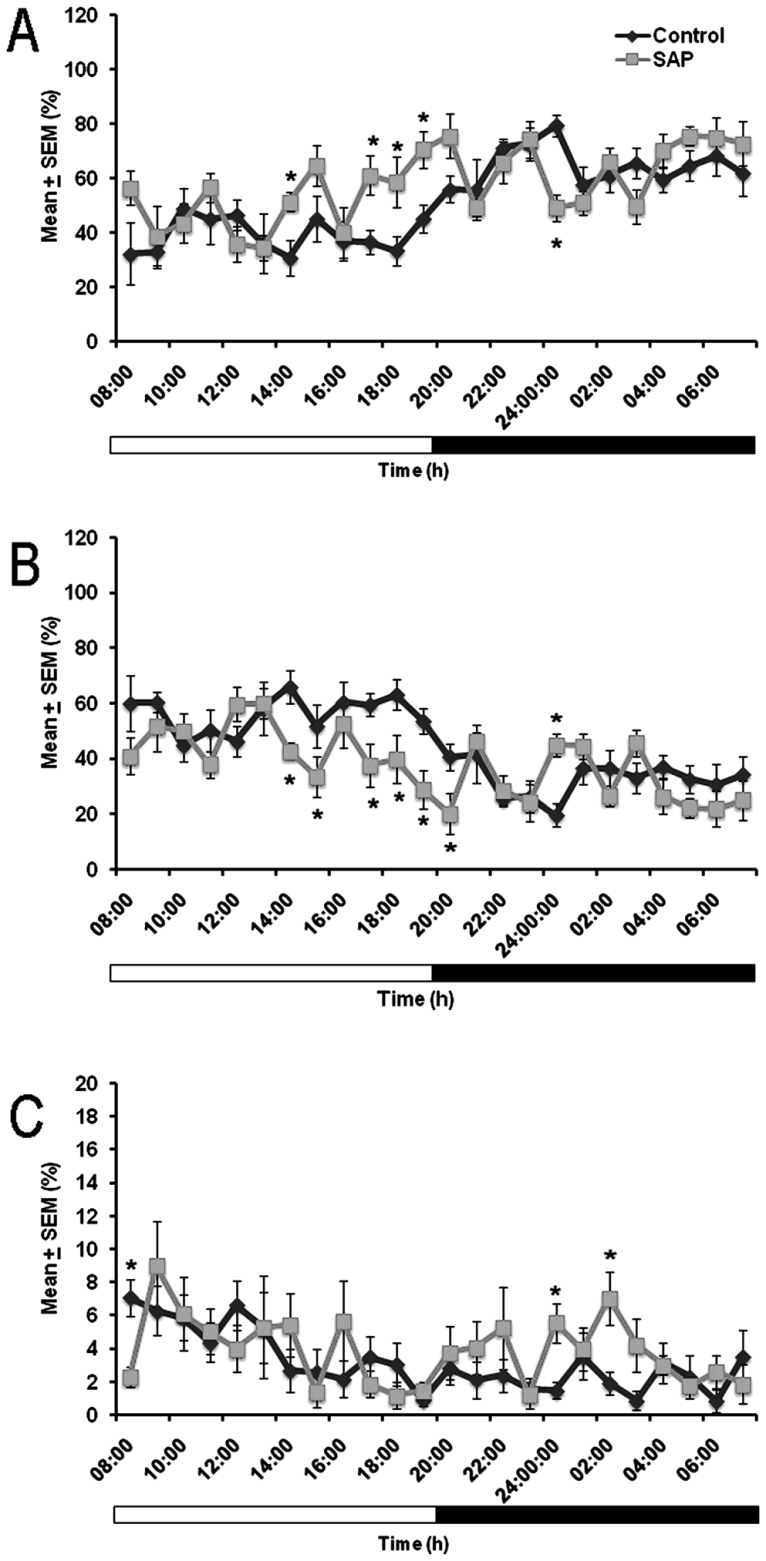
The hourly data at 21 days post-grafting HCRT neurons into LH of saline solution or HCRT2/SAP lesioned animals for wakefulness (W) (Panel A), slow wave sleep (SWS) (Panel B) and rapid eye movement sleep (REMS). The white bars represent the lights-on period and the black bars indicate the lights-off period (Panel C; mean ± SEM of total time of recording [%]; * *vs* control, p<0.05).

**Fig. 4** shows the hourly analysis of the sleep-wake cycle across 24 h. In the control and HCRT2/SAP lesioned groups at day 21 post-transplant, the hourly analysis of W (Panel A) showed statistical differences at the following hours: 7^th^ (df = 14; *t* = −2.746; *p*<0.01), 10^th^ (df = 5; *t* = −2.874; *p*<0.01), 11^th^ (df = 14; *t* = −2.327; *p*<0.03), 12^th^ (df = 14; *t* = −3.013; *p*<0.009), and 17^th^ (df = 14; *t* = 4.827; *p*<0.0003). Hourly analysis of SWS (Panel B) showed that lesioned rats that received HCRT graft displayed a significant enhancement during the lights-on period, compared to control group that also was transplanted with HCRT neurons, at the following hours: 7^th^ (df = 14; *t* = 3.427; *p*<0.004), 8^th^ (df = 14; *t* = 1.707; *p*<0.1), 10^th^ (df = 14; *t* = 2.475; *p*<0.02), 11^th^ (df = 14; *t* = 2.248; *p*<0.04), 12^th^ (df = 14; *t* = 2.977; *p*<0.01), 13^th^ (df = 14; *t* = 2.309; *p*<0.03), and 17^th^ (df = 14; *t* = −4.267; *p*<0.0008). Finally, the hour by hour analysis of REMS (Panel C) showed that lesioned animals displayed an enhancement at the 1^st^ (df = 14; *t* = 1.208; *p*<0.05), 17^th^ (df = 14; *t* = −3.544; *p*<0.003), and 19^th^ (df = 14; *t* = −2.791; *p*<0.01) hours compared to control group.

## Discussion

Narcolepsy is a neurodegenerative disorder characterized by excessive daytime sleepiness and typically associated with cataplexy. This disease is extremely incapacitating, and results in impaired psychosocial functioning and reduced work performance [25], [31]–[33]. The neurobiological role of HCRT system in the control of alertness has been demonstrated in association with the onset of narcolepsy. Diverse experimental narcoleptic animal models have been developed in order to understand the neurophysiological processes of narcolepsy. The present study shows that, among these diverse models, the injection of HCRT2/SAP into the LH of rats diminishes the number of HCRT neurons and induces a narcoleptic-like behavior. These results are in accordance with previous reports [9], [26]–[28]. Furthermore, the administration of this toxin diminished W, and increased SWS and REMS during the lights-off period. The enhancement of sleepiness observed in our study is also reported in narcoleptic humans [34].

Due to the fact that we faced methodological challenges such as the time of permanence of the electrodes on the skull (animals were losing the sleep-electrodes implants), the number of rats successfully studied in the current report was small and it may be difficult to elaborate strong claims based on this result. Therefore, we consider the findings as a preliminary result. The procedure of transplants may have major importance in long term studies since the improvement in sleep was observed at 21 days post-transplant. The grafts were able to improve the excessive somnolence observed in the narcoleptic-like model. Although waking was enhanced and SWS diminished during the lights-on period and enhanced SWS after grafting as seen in the hourly analysis.

Despite the fact that the number of HCRT neurons in lesioned rats after grafting was not provided it is highly possible that a significant number of HCRT grafted neurons in lesioned rats could survive and were able to provide the neurophysiological conditions for a behavioral improvement. The prior studies published by our group may support this assumption. For instance, the number of HCRT transplanted neurons decreases significantly after grafting, however a percentage of this neurons do survive until day 36 [23], [24]. Regarding this, it is known that in the cell transplant strategies, as reported in Parkinson's disease, a relatively small number of surviving neurons produce the recovery of function in animals and in humans [35]–[38]. But, how might one determine the functional viability of the surviving HCRT neurons in our study? One possibility is to describe whether the graft releases the peptide; however, this might be difficult since the amount of ligand released by the relatively small number of surviving HCRT neurons could not be detectable using current methods even though some HCRT neurons might still be present in the LH. This scenario is the case in human narcolepsy since the peptide is undetectable in the CSF. Furthermore, in rats it has been reported that 70% loss of HCRT neurons induces 50% decline in HCRT levels measured in CSF [28].

For instance, the use of intranasal administration of HCRT-1 diminishes REMS in narcoleptic patients and represents an interesting perspective [39]–[41]. Our most prudent assessment is that the data presented in our study suggests that exploring the cell transplant technique could be considered to treat narcolepsy. This method, however, has limitations: (i) the source of orexinergic neurons is from postnatal tissue and (ii) the survival rate of the grafts is very poor (∼5% of implanted cells) [3], [23], [24]. The main interest is now focused on producing orexinergic neuroblasts for transplantation from stem cells. After maturation, these neurons have to work at least as well as those living in the adult LH. Conceivably, the stem cell-derived cells have to fulfill the following requirements to induce recovery from narcolepsy symptoms after transplantation: (1) higher graft survival probability; (2) release orexins in a regulated manner; (3) exhibit the molecular, morphological and electrophysiological properties of fully mature orexinergic neurons; (4) re-establish a dense, functional orexin releasing terminal network; (5) grafts have to become functionally integrated into host circuitries to restore wakefulness, for example. The human application of stem cell-derived orexinergic neuroblasts will be based on solid preclinical work in animal models of the disease.
